# Understanding sexual dysfunction in french military service member with PTSD: findings from a descriptive study

**DOI:** 10.1186/s12610-025-00266-1

**Published:** 2025-05-26

**Authors:** Gilles Sipahimalani, Marion Remadi, Antoine Baldacci, Thibaut Long-Depaquit, Arthur Peyrottes, Marie Dusaud, Emeric Saguin

**Affiliations:** 1 Psychiatry Department, Hôpital National Des Armées Percy, Clamart, France; 2 Psychiatry Department, Hôpital National Des Armées Bégin, Saint-Mandé, France; 3 Urology Department, Hôpital National Des Armées Saint-Anne, Toulon, France; 4 Urology Department, Hôpital Saint-Louis, Paris, France; 5 Urology Department, Hôpital National Des Armées Bégin, Saint-Mandé, France; 6 VIFASOM (Vigilance Fatigue Sommeil Et Santé Publique) URP 7330, Université Paris Cité (Paris), Paris, France

**Keywords:** Post-traumatic stress disorder, Sexual dysfunction, Sexual health, Military personnel, Trouble de Stress post-traumatique, Dysfonction sexuelle, Santé sexuelle, Personnel militaire

## Abstract

**Background:**

Sexual dysfunction is a serious and frequently underrecognized consequence of Post-Traumatic Stress Disorder (PTSD), with profound implications on quality of life. This study aimed to characterize sexual dysfunction observed in French military personnel suffering from PTSD.

**Results:**

This retrospective study included 32 male French military personnel diagnosed with PTSD who participated in a sexual dysfunction screening program at a French National Military Hospital between October 2023 and August 2024. The mean age of participants was 40 years. The Post-Traumatic Stress Disorder Checklist Scale version DSM-5 (PCL-5) and the International Index of Erectile Function version 5 (IIEF-5) were used to assess PTSD and sexual dysfunction severity.

Seventy-two percent (*n* = 23) reported at least one sexual dysfunction, with sexual desire dysfunction being the most common sexual health issue. Approximately 70% perceived the impact of sexual dysfunction on quality of life as high or very high. Significant correlations were found between PCL-5 and IIEF-5 scores, suggesting that the severity of PTSD symptoms impacts sexual health, specifically concerning cluster B (re-experiencing symptoms) and cluster D (negative thoughts and/or feelings) symptoms. Psychiatric symptoms were the primary risk factor, followed by psychotropic treatment and personal factors.

**Conclusion:**

This study highlights the high prevalence of sexual dysfunction among French military personnel with PTSD. The diversity of the clinical presentations and risk factors emphasize the need for comprehensive sexual health assessments in PTSD management. Personalized care strategies targeting both psychiatric and sexual health issues are essential for improving outcomes in this population.

**Supplementary Information:**

The online version contains supplementary material available at 10.1186/s12610-025-00266-1.

## Résumé

**Contexte:**

La dysfonction sexuelle est une conséquence grave et souvent sous-estimée du trouble de stress post-traumatique (TSPT), avec de profondes implications sur la qualité de vie. Cette étude visait à caractériser les dysfonctionnements sexuels observés chez les militaires français souffrant de TSPT.

**Résultats:**

Cette étude rétrospective a porté sur 32 militaires français, de sexe masculin, diagnostiqués avec un TSPT, qui ont participé à un programme de dépistage de la dysfonction sexuelle dans un hôpital national militaire français entre octobre 2023 et août 2024. L’âge moyen des participants était de 40 ans. L’échelle de contrôle du trouble de stress post-traumatique version DSM-5 (PCL-5) et l’Indice international de la fonction érectile version 5 (IIEF-5) ont été utilisés pour évaluer la gravité du TSPT et de la dysfonction sexuelle.

Soixante-douze pour cent (n = 23) ont signalé au moins une dysfonction sexuelle, la dysfonction du désir sexuel étant le problème de santé sexuelle le plus courant. Environ 70% percevaient l’impact de la dysfonction sexuelle sur la qualité de vie comme élevé ou très élevé. Des corrélations significatives ont été trouvées entre les scores PCL-5 et IIEF-5, suggérant que la gravité des symptômes du TSPT a un impact sur la santé sexuelle, en particulier en ce qui concerne les symptômes du groupe B (réapparition des symptômes) et du groupe D (pensées et/ou sentiments négatifs). Les symptômes psychiatriques étaient le principal facteur de risque, suivis du traitement psychotrope et des facteurs personnels.

**Conclusions:**

Cette étude met en évidence la forte prévalence de dysfonction sexuelle chez les militaires français atteints de TSPT. La diversité des présentations cliniques et les facteurs de risque soulignent la nécessité d’évaluations complètes de la santé sexuelle dans la prise en charge du TSPT. Des stratégies de soins personnalisées ciblant à la fois les problèmes de santé psychiatrique et sexuelle sont essentielles pour améliorer les résultats dans cette population.

## Introduction

Post-Traumatic Stress Disorder (PTSD) is a significant and enduring health concern among military personnel, with profound implications for mental health, quality of life, and operational readiness. French military populations, exposed to repeated trauma during deployments, experience PTSD at rates ranging from 1.7% to 8% [1–3]. The disorder manifests in four core symptom clusters: re-experiencing, hypervigilance, avoidance, and negative alterations in cognition and mood [4]. Beyond its psychological impact, PTSD disrupts personal, family, and social functioning, often accompanied by psychiatric, physical, and addictive comorbidities that further degrade quality of life [5, 6].

Sexual dysfunction is a common but under-recognized consequence of PTSD. Prevalence estimates in military populations vary widely, from 8.4% to 88.6%, with erectile dysfunction and reduced sexual desire being the most frequently reported [7]. The mechanisms underlying this association remain multifactorial and are not yet fully elucidated. Biologically, they involve complex neuroendocrine disruptions, which may contribute to alterations in sexual function. On the cognitive and behavioral level, factors such as anxiety-induced hypervigilance and maladaptive coping strategies likely exacerbate the condition. Interpersonally, the dynamics of impaired communication and strained relationships could further complicate the experience of sexual dysfunction. Psychiatric comorbidities, such as depression and trauma-related disorders, are frequently observed and may significantly influence the severity of sexual dysfunction [7, 8]. Additionally, substance use disorders and somatic conditions, such as cardiovascular disease, exacerbate the risk of dysfunction [7, 9, 10]. Physical activity may act as a protective factor [10], while psychotropic medications, particularly antidepressants, often contribute to sexual side effects [11], though they may alleviate dysfunction in some cases [12].

Despite its profound impact on quality of life [13], sexual dysfunction remains a neglected aspect of PTSD management in military settings, due to cultural stigma [14] and healthcare provider hesitancy [15]. This study aims to explore the prevalence, characteristics, and contributing factors of sexual dysfunction in military personnel with PTSD, highlighting the need for comprehensive care strategies.

## Material and methods

### Sexual health issue screening program

To improve the management of sexual dysfunction in the military with PTSD, a screening program was implemented in the psychiatry department of a French Military Training Hospital. Developed in collaboration with the urology department, this program systematically assesses sexual issues, risk and prognostic factors, quality-of-life impacts, and comorbidities. It includes a dedicated questionnaire (see Additional file 1) and two validated self-report measures: the Post-Traumatic Stress Disorder Checklist Scale version DSM-5 (PCL-5) [16], which evaluates PTSD symptoms, and the International Index of Erectile Function version 5 (IIEF-5) [17], which assesses erectile function.

### Aims

This study aimed to describe the results of the screening program for sexual dysfunction. The primary objective was to measure the prevalence of sexual dysfunction in this population. Additionally, three secondary objectives were outlined: to identify the specific types of sexual dysfunctions observed, to provide an analytical framework for understanding their progression, and to investigate potential cofactors associated with these dysfunctions.

### Study design and population

This descriptive, retrospective, single-center study was conducted in the psychiatry department of a French Military Training Hospital. Referring psychiatrists identified patients (hospitalized and outpatients) who had undergone sexual dysfunction screening since the screening program's implementation in October 2023. The questionnaires were completed during a consultation with the referring psychiatrist. The systematic screening could be conducted at the start or during medical follow-up of any patient with PTSD. Eligible subjects were screened patients who provided consent for the use of their medical data for research purposes.

### Data collection

Sociodemographic and clinical data were extracted from the electronic medical records of each included patient. Sociodemographic variables included age, sex, marital status, and service history. Clinical data comprised information on psychiatric, addictive, and somatic comorbidities, psychotropic treatments, and levels of physical activity.

Data related to sexual dysfunction were collected using a questionnaire developed by a multidisciplinary team of psychiatry and urology experts as part of the screening program. The latter included details on sexual health issues, temporal patterns and associated factors, the impact of dysfunction on quality of life, and treatments received. In addition, the results of the PCL-5 and the IIEF-5 were integrated.

### Statistical analysis

Continuous variables were reported as means with standard deviation, while categorical variables were presented as percentages with raw numbers.

Sexual function was evaluated using three parameters: the total score on the IIEF-5, and its two sub-scores (erectile function assessed with questions 1–4 and sexual satisfaction assessed with question 5). The negative impact of sexual dysfunction on quality of life was rated on a Likert scale within the screening questionnaire, ranging from 0 (no impact) to 5 (very high impact).

PTSD symptoms were assessed using the PCL-5, which evaluates five dimensions: overall severity (total score), re-experiencing syndrome (Cluster B, questions 1–5), avoidance syndrome (Cluster C, questions 6 and 7), negative cognitions and emotions (Cluster D, questions 8–14), and hypervigilance syndrome (Cluster E, questions 15–20).

Finally, Spearman’s rank test was used to test the correlation analyses significancy. Tests were considered statistically significant with p values of < 0.05. Statistical analyses were conducted using Jamovi software (Version 2.4) [18].

## Results

### Sociodemographic and clinical characteristics

A total of 32 subjects diagnosed with PTSD were included in the sexual dysfunction screening program between October 2023 and August 2024. All subjects were male. Among the subjects, 46.9% (*n* = 15) had at least one psychiatric comorbidity including major depressive disorder (37.5%; *n* = 12) and general anxiety disorder (9.4%, *n* = 3), 59.4% (*n* = 19) presented one or more substance use disorders and 53.1% (*n* = 17) reported at least one medical comorbidity or surgical history. Those included cardiovascular diseases (*n* = 5), neurological diseases (*n* = 5), endocrine diseases (*n* = 3), urological diseases (*n* = 4) and orthopedic history (*n* = 5). The sociodemographic and clinical characteristics are reported in Table 1.

**Table 1:** Sociodemographic and clinical characteristics

	**Sample (*n* = 32)**
**Sociodemographic data**	
**Age (years)**	40.0 (9.31)
**Marital status**	
Married	34.4% (11)
Coupled	31.3% (10)
Single	34.4% (11)
**Children**	65.6% (21)
**Military history**	
**Years of duty**	17.6 (8.25)
**Number of deployments**	6.06 (4.83)
**Physical activity**	64.5% (20)
**Substance use disorders**	
Tobacco	50.0% (16)
Alcohol	25.0% (8)
Cannabis	9.4% (3)
**Psychotropic drugs**	
**Psychotropic drugs prescribed**	79.3% (23)
**Number of drugs (if prescribed)**	2.28 (1.21)
**Types of drugs**	
Antidepressants	74.1% (20)
First-generation antipsychotic	59.3% (16)
Second-generation antipsychotic	37.0% (10)
Benzodiazepines	7.4% (2)
**PTSD symptoms (PCL-5)**	
Total score	42.8 (15.5)
Cluster B (re-experiencing symptoms)	17.2 (12.9)
Cluster C (avoidance symptoms)	4.48 (2.69)
Cluster D (negative thoughts and/or feelings)	14.5 (5.45)
Cluster E (hyperarousal and reactivity)	12.4 (5.38)

*PCL-5* Post-traumatic disorder CheckList scale version DSM-5, *PTSD* Post-traumatic Stress Disorder, *TBI* Traumatic Brain Injury

Results are presented as means (standard deviation) or percentages (number of cases)

### Frequency and typology of sexual dysfunctions

**Fig. 1:**
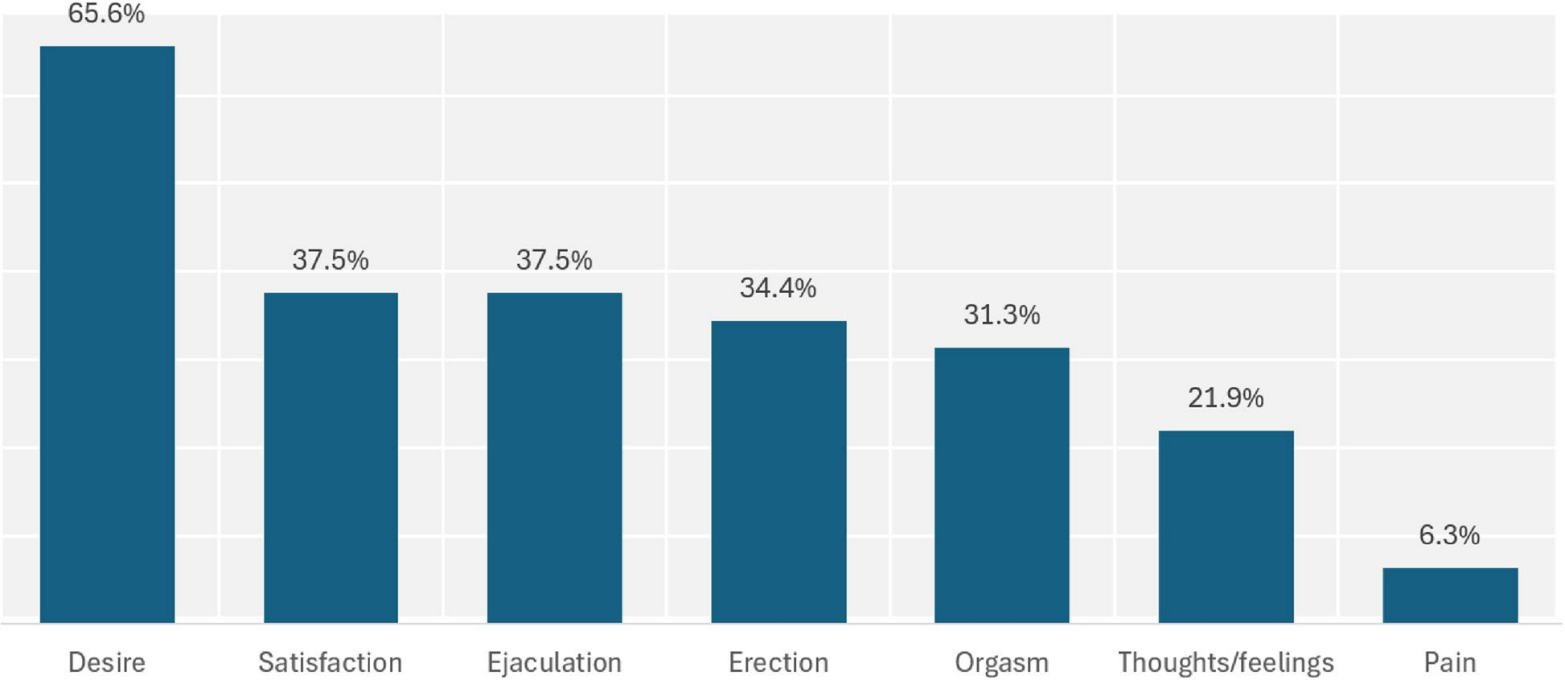
Frequency and types of reported sexual health issues

Among the subjects, 71.9% (*n* = 23) reported one or several issues related to sexual function, with an average number of issues of 3.26 (SD 1.76). Among the subjects with no issues at the time of inclusion, 37.5% (*n* = 3) reported having experienced sexual dysfunction in the past. The IIEF-5 identified erectile dysfunction in 48.4% (*n* = 15) of the subjects (Table 2). Sexual health results are shown in Fig. 1.

**Table 2:** Sexual health issue assessed with the IIEF-5

	**Sample (*n* = 32)**
**Total score**	15.9 (7.30)
**Erectile function (questions 1–4)**	13.0 (5.91)
**Satisfaction (question 5)**	2.87 (1.63)
**Scoring**	
No erectile dysfunction	41.9% (13)
Mild erectile dysfunction	12.9% (4)
Moderate erectile dysfunction	25.8% (8)
Severe erectile dysfunction	9.7% (3)
Uninterpretable	9.7% (3)

*IIEF-5* International Index of Erectile Function version 5

Results are presented as means (standard deviation) or percentages (number of cases)

Among the subjects reporting sexual health issues, 68.4% (*n* = 13) identified sexual desire dysfunction as their primary issue, followed by ejaculation dysfunction (15.8%, *n* = 3), erectile dysfunction (10.5%, *n* = 2), and negative thoughts and emotions related to sexual function (5.3%, *n* = 1).

Except for one patient describing increased sexual desire, sexual desire dysfunction was characterized by a decrease or total absence of sexual desire. Two patients specified the nature of this dysfunction: one explained that he felt overly sedated, while the other described feeling"disconnected from his emotions."Erectile dysfunction consisted of a decrease or absence of erections. Orgasm or ejaculation abnormalities most often involved delayed or absent orgasm/ejaculation, though two patients described premature ejaculation.

Sexual satisfaction and associated cognitions/emotions led to several comments. Four subjects reported feelings of"guilt,""embarrassment,"and/or"sadness"towards their partner, associated with decreased self-esteem and rumination about"their performance."Two subjects described pain during sexual activity. One reported chronic perineal pain linked to a physical trauma, exacerbated during sexual activity. The other reported pain localized to the glans, associated with mucosal dryness since starting an antidepressant.

Sexual activity was rated as moderately to highly important, with an average score of 3.57 (SD = 0.99). Nearly 10% rated this as low, very low, or nonexistent, while 40% rated it as moderate, and 50% rated it as high or very high. The impact of sexual dysfunctions on quality of life was rated at 3.88 (SD = 1.58, between moderate and high on the Likert scale), ranging from moderate to high. Approximately 70% considered this impact to be high or very high, 10% moderate, and 20% low, very low, or nonexistent.

Sexual dysfunctions led to a targeted adjustment in management in 56.5% (*n* = 13) of cases, with various approaches including: prescription of tadalafil (*n* = 8), treatment interruption (*n* = 1), increasing the time between psychotropic medication administration and sexual intercourse (*n* = 1), initiation of antidepressant treatment (*n *= 1), and management of benign prostatic hyperplasia (*n* = 1). Two subjects were referred for urological consultation.

### Analytical approach to sexual dysfunctions and relationship with PTSD symptoms

**Fig. 2:**
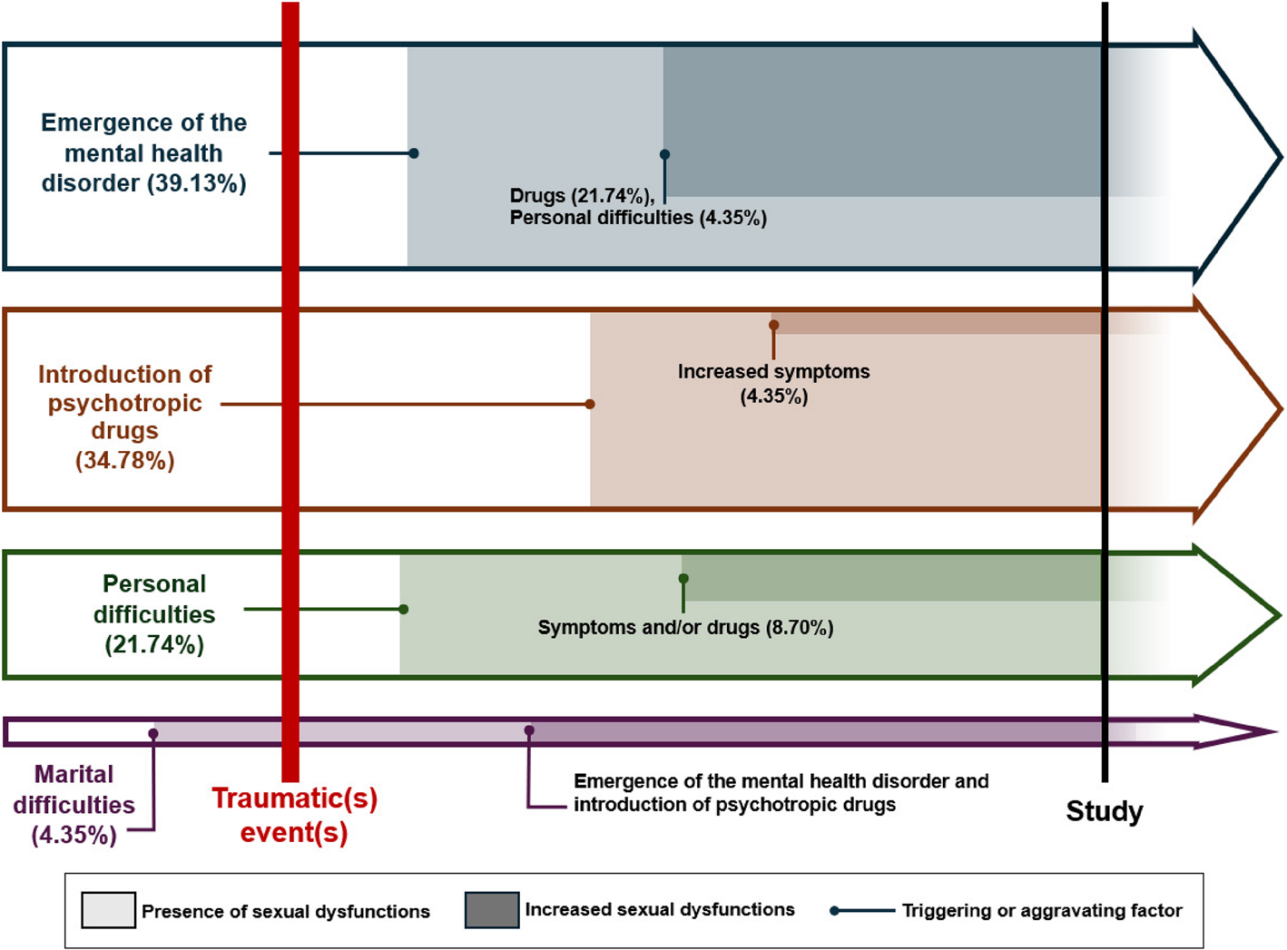
Chronological approach to sexual health issues factors

Ninety-six percents (*n* = 22) of patients with sexual dysfunction reported that the onset of the dysfunction occurred after the psychological trauma. The emergence of the dysfunctions was linked to the onset of post-traumatic symptoms or their decompensation in 40.9% (*n* = 9) of cases, the initiation of psychotropic treatment in 36.4% (*n* = 8) of cases, and personal, marital, or family factors in 22.7% (*n* = 5) of cases. The most implicated treatments were selective serotonin reuptake inhibitors (SSRIs), particularly sertraline, and antipsychotics (including quetiapine and cyamemazine). Contributing personal factors included marital difficulties (*n* = 3), the birth of a child (*n* = 1), and retirement (*n* = 1). The only subject who reported sexual health issues before the traumatic event identified marital difficulties as the triggering factor. The evolution of sexual dysfunctions based on triggering and aggravating factors is shown in Fig. 2.

**Fig. 3:**
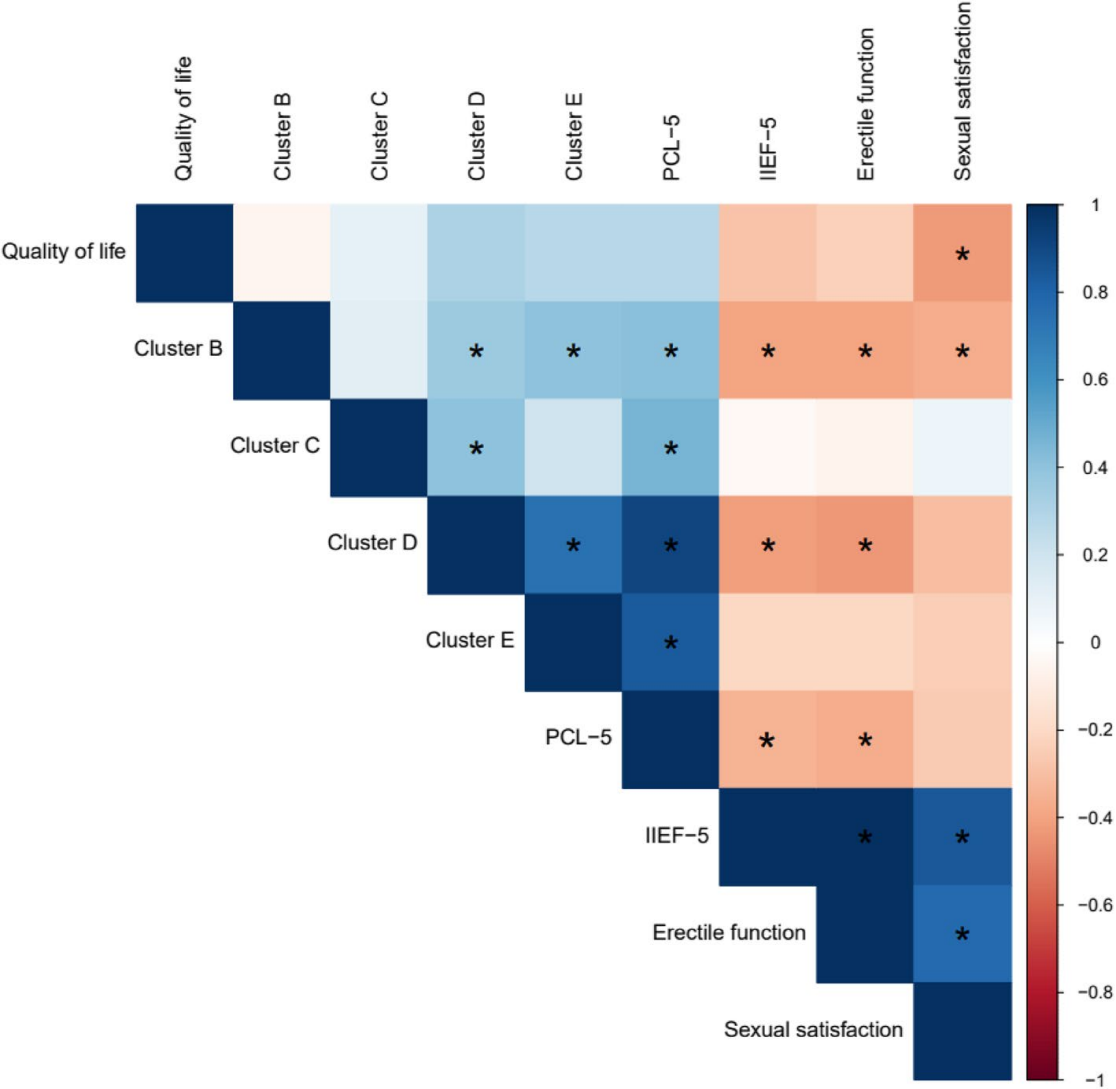
Correlation between sexual dysfunction and PTSD symptoms

Correlations analyses identified significant associations between sexual function parameters and PTSD symptoms (Fig. 3). Erectile dysfunction and sexual satisfaction were associated with higher PCL-5 scores, particularly in the subscales for intrusion syndrome and negative changes in cognitions and emotions.

The impact on quality of life was correlated with the IIEF-5 score and erectile function. Age was significantly correlated with erectile function (Spearman's Rho = −0.390; *p* = 0.03), but not with the total IIEF-5 score or sexual satisfaction. The associations between PCL-5 scores and sexual parameters remained significant after adjusting for age. Post-hoc analyses showed that the number of psychotropic medications, the number of hours of exercise per week, and the number of pack-years of smoking showed no significant association with sexual function parameters.

## Discussion

This is the first known investigation to examine sexual function impairments in French military personnel suffering from PTSD. In our sample, nearly 70% of participants reported at least one sexual health issue, and almost 50% exceeded the pathological threshold on the IIEF-5. The most common issue was a decrease or even abolition of sexual desire. The analytical approach revealed that sexual dysfunctions emerged concurrently with the onset of post-traumatic symptoms in nearly 40% of cases, were associated with the initiation of psychotropic treatment in 35% of cases and were attributed to individual psychosocial stressors in 25% of cases. Correlation analyses suggested a link between sexual function impairments and PTSD symptoms, particularly with the re-experiencing syndrome and negative cognitive and emotional disturbances. This reinforces the association between core PTSD symptoms and sexual dysfunction, further supported by the strong correlation between the severity of dysfunction and its negative impact on quality of life.

The association between the severity of PTSD symptoms and sexual dysfunctions leads to several mechanistic hypotheses. Some authors suggest that PTSD patients may avoid sexual intercourse to limit their sense of vulnerability and hypervigilance manifestations. Evening hours, often marked by anticipatory anxiety about nocturnal flashbacks, may further exacerbate avoidance behaviors. Cognitive and emotional alterations (cluster D [4]) have shown a correlation with sexual dysfunctions, suggesting a possible impact of emotional numbing [19]. Characterological changes, such as irritability or even hostility, often reported by veterans with PTSD, contribute to interpersonal difficulties and may affect intimate relationships [9]. In our sample, the correlation between sexual dysfunction and re-experiencing syndrome or negative emotional disturbances supports the hypothesis that anticipatory anxiety (e.g., fear of flashbacks) and emotional blunting may underlie these dysfunctions.

Biologically, neuroendocrine dysfunctions involving the hypothalamo-pituitary axis and the autonomic nervous system could alter the regulation of sex hormones [20]. Among veterans with PTSD, sexual dysfunction has been associated with altered levels of plasma dehydroepiandrosterone, cortisol, urinary catecholamines, and glucocorticoid sensitivity, independently of comorbid depression [21]. These hormonal abnormalities may be considered within a broader and more complex clinical framework, accounting for the comorbidities frequently associated with PTSD, such as traumatic brain injury and polytrauma involving the urogenital system [22], or, alternatively, metabolic conditions like diabetes and hypertension [19]—each of which is known to be associated with sexual dysfunction. Mechanistic hypotheses suggest that PTSD-related hormonal dysregulation may interfere with pathways involved in sexual function [23]. However, the underlying mechanisms remain insufficiently elucidated, highlighting the need for ongoing multidisciplinary research. Nonetheless, mechanistic approaches should not eclipse the patient’s subjective experience, which remains central to patient-centered clinical care.

Various therapeutic approaches have been employed to address PTSD. Despite significant clinical efforts, current treatments yield only partial remission [24, 25]. While one-third of patients with acute post-traumatic symptoms show a favorable course within six months, the majority continue to experience symptoms for several years, with an increased risk of social and professional disengagement and suicide [26–29]. Sexual dysfunctions in veterans with PTSD may persist despite trauma-focused treatment and often require targeted interventions. Interpersonal relationships and coping strategies may influence the relationship between PTSD and sexual dysfunction. Marital dynamics could exacerbate or mitigate dysfunctions, with structured couple therapy offering a potential avenue for intervention [30]. Recommended approaches include sex therapy, which focuses on restructuring dysfunctional beliefs and reducing performance anxiety, as well as sensate focus exercises to restore intimacy. Pharmacological treatments may be considered for physiological symptoms. Psychoeducation is also essential, helping individuals understand the impact of trauma on sexuality and normalize their experiences. Integrating these strategies can improve outcomes and support a more holistic recovery [9]. Additionally, attitudes toward sexuality and individual resilience may play moderating roles that warrant further exploration.

Including female personnel in future research is essential, as sexual dysfunctions in women with PTSD may involve distinct mechanisms and clinical manifestations, particularly those potentially linked to sexual assault trauma. PTSD may exacerbate the severity of sexual dysfunctions that could emerge independently following such events. Avoidance of sexual activity is frequently observed among survivors of sexual assault [31]. Biologically, the hormonal milieu in women differs markedly from that of men, which may contribute to sex-specific vulnerabilities. Furthermore, psychotropic treatments may be linked to distinct adverse effects in women, including more pronounced reductions in sexual desire and higher rates of anorgasmia compared to men [32].

Approximately two-thirds of the subjects reporting sexual health issues demonstrated sexual desire dysfunction. Sexual desire can be defined as the motivational component of sexual activity [33]. The association between PTSD and alterations in sexual desire has been extensively documented [8], as well as its correlation with comorbid depression. Furthermore, it has been suggested that sexual desire may improve alongside the reduction of PTSD symptoms [34]. The clinical implications of this study highlight the utility of the current screening program, despite its limitations. A 30% discrepancy between patient-reported sexual health issues and IIEF-5 findings suggests the need for refinement. Sexual desire dysfunctions, which were prevalent in this sample, are not adequately captured by the IIEF-5. Enhancing the screening program to include additional dimensions of sexual health, particularly desire-related issues, could improve its accuracy.

Among psychiatrists, barriers to addressing sexual health often include the perception that “a different problem was more important,” “lack of time,” “embarrassment” [35]. These findings, however, have yet to be replicated in the population of military healthcare providers. Although sexual health is sometimes considered secondary in clinical prioritization, findings from this study suggest that individuals with PTSD in military settings may regard it as a significant component of their quality of life. Addressing these barriers through targeted training for clinicians, along with the larger implementation of screening tools, could help raise awareness of the issue, while also providing support and reassurance to professionals in their clinical practice. Integrating sexual health assessments into routine psychiatric care could help reduce stigma and improve the detection and management of sexual dysfunction in military populations [36].

Pharmacological management of PTSD-related sexual dysfunction should follow a personalized approach. In 40% of cases, the onset of psychotrauma symptoms was identified as a risk factor for sexual dysfunction, potentially leading to the introduction of antidepressant treatment [12]. Conversely, nearly 35% of participants linked their sexual dysfunction to the initiation of psychotropic treatment. This underscores the need for careful evaluation of medication regimens and the potential role of pharmacogenetic protocols in minimizing side effects [37, 38]. Symptomatic treatments, such as tadalafil [39], could improve erectile function, and referrals to urologists should be considered for complex cases. Addressing comorbidities, such as substance use disorders or somatic conditions, may also improve sexual health outcomes. Ongoing research in this field within the department aims to develop our knowledge and create more individualized care pathways.

### Study limitations

This study also highlights the limitations inherent to its design. The retrospective, self-reported nature of the dataset introduces potential selection and recall biases. As the study examines existing information without prospective tracking, it is unable to establish a clear temporal sequence. This issue underscores the need for future prospective studies that could more effectively establish causal relationships and explore the directionality of these associations.

The screening process, while effective for initial detection, lacks the depth of a more comprehensive clinical evaluation, and paraclinical investigations were not performed. Recruitment was based on PTSD diagnoses made by the referring psychiatrist, which may impact the representativeness of the sample. Another limitation is that psychotherapeutic interventions were not considered in the Sexual Health screening, preventing control of their potential impact. Given that these interventions are a gold standard in PTSD treatment and can affect sexual function [40], future research should account for them to better assess their influence on sexual health in PTSD patients. Due to the limited sample size, a power analysis was not performed. This constitutes a limitation, as the study may be underpowered to detect small to moderate effects. Additionally, the study exclusively included male subjects, reflecting the overrepresentation of men in military populations. Future studies could include with larger samples, standardized diagnostic tools for PTSD and systematically recruit both male and female personnel to explore potential gender-specific differences in sexual dysfunction.

## Conclusion

This study highlights a high prevalence of sexual dysfunctions in French military personnel suffering from PTSD. Their pathophysiological mechanisms and risk factors remain poorly understood, stressing the need for researchers to focus on this topic. A study is soon expected to evaluate the perspective of French military healthcare professionals on sexual dysfunction. The next steps will involve developing recommendations, establishing a multidisciplinary care pathway, and training practitioners on sexual health issues, with the aim of improving the quality of life of military personnel with PTSD.

## Supplementary Information

Additional file 1. Sexual health issues screening questionnaire (pdf).

## Data Availability

No datasets were generated or analysed during the current study.

## Abbreviations

PTSD: Post-Traumatic Stress Disorder
PCL-5: Post-Traumatic Stress Disorder Checklist Scale version DSM-5
IIEF-5: International Index of Erectile Function version 5
