# Supplementary material for: Understanding sexual dysfunction in french military service member with PTSD: findings from a descriptive study
Source: Basic Clin Androl. 2025 May 26;35:19. doi: 10.1186/s12610-025-00266-1 (PMC12105166; doi:10.1186/s12610-025-00266-1)
Supplement: Supplementary file 1 — Additional file 1. Sexual health issues screening questionnaire (pdf). [file 12610_2025_266_MOESM1_ESM.pdf]

Formulaire de dépistage et d'évaluation des troubles de la fonction sexuelle

Date :

Nom : Prénom : Date de naissance : / /

Diagnostic psychiatrique principal :

☐ ESPT ☐ Trouble dépressif ☐ Trouble anxieux ☐ Autre, préciser : ....

Comorbidités psychiatriques :

☐ ESPT ☐ Trouble dépressif ☐ Trouble anxieux ☐ Autre préciser : ....

Comorbidités addictives

☐ Tabac
Paquets-années : ......
☐ Trouble de l'usage de l'alcool ☐ Trouble de l'usage du cannabis

☐ Autre(s), préciser : ....

Comorbidités physiques :

☐ Cardiovasculaires
Préciser : ....
☐ Neurologiques
Préciser : ....
☐ Traumatiques
Préciser : ....
☐ Uro/gynécologique: ....

☐ Endocrinologiques : .... ☐ Autres.............................

Pratique sportive (nombre d'heures par semaine) : ....

Traitement actuel (y compris traitements psychotropes) :

....

....

....

....

....

....

Plainte(s) relative(s) à la fonction sexuelle : ☐ OUI ☐ NON

Si oui, altération de :

☐ Excitation sexuelle (libido), préciser : ....

☐ Capacité à atteindre/maintenir une érection, préciser : ....

☐ Capacité à éjaculer, préciser : ....

☐ Capacité à atteindre un orgasme, préciser : ....

☐ Satisfaction sexuelle, préciser : ....

☐ Présence de douleurs liées à l'activité sexuelle, préciser : ....

☐ Présence de cognitions/émotions négatives liées à l'activité sexuelle, préciser : ....

☐ Autre(s), préciser ....

→ **Souligner la plainte principale**

Temporalité des troubles de la fonction sexuelle

Indiquer sur la frise :

- La date du traumatisme et la date du déclenchement des symptômes psychotraumatiques ;
- La date du début des difficultés sexuelles (griser la période concernée par les troubles);
- Les évènements et autres facteurs identifiés (rupture amoureuse, AVP, introduction d'un ISRS, …).

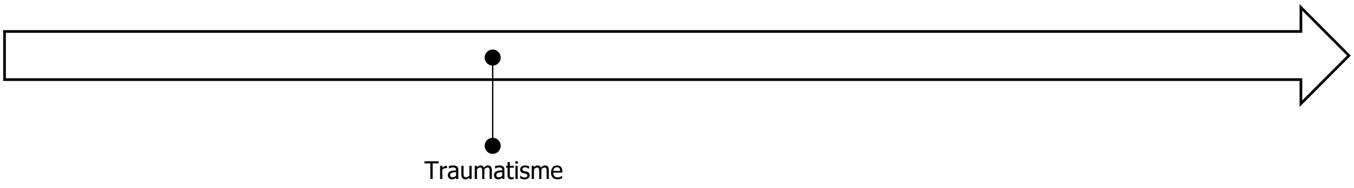

Importance accordée à l'activité sexuelle dans le quotidien (entourer) :

**0**	**1**	**2**	**3**	**4**	**5**
Aucune	Très faible	Faible	Modérée	Elevée	Très élevée

Altération de la qualité de vie liée aux dysfonctions sexuelles (entourer) **:**

**0**	**1**	**2**	**3**	**4**	**5**
Aucune	Très faible	Faible	Modérée	Elevée	Très élevée

Prise en charge proposée (le cas échéant) : ....

Le patient souhaite-t-il bénéficier d'un rendez-vous en urologie ? ☐ OUI ☐ NON
